# Validation of a leg movements count and periodic leg movements analysis in a custom polysomnography system

**DOI:** 10.1186/s12883-017-0821-6

**Published:** 2017-02-23

**Authors:** Ambra Stefani, Anna Heidbreder, Heinz Hackner, Birgit Högl

**Affiliations:** Department of Neurology, Medical University of Innsbruck, Anichstrasse 35, Innsbruck, A-6020 Austria

**Keywords:** PLM, Restless legs syndrome, Sleep, PSG, Computerized scoring

## Abstract

**Background:**

Periodic leg movements (PLM) during sleep (PLMS) are considered strongly related to restless legs syndrome (RLS), and are associated with polymorphisms in RLS risk genes. Various software for automatic analysis of PLMS are available, but only few of them have been validated. Aim of this study was to validate a leg movements count and analysis integrated in a commercially available polysomnography (PSG) system against manual scoring.

**Methods:**

Twenty RLS patients with a PLMS index > 20/h and 20 controls with a PLMS index < 5/h were included. Manual and computerized scoring of leg movements (LM) and PLM was performed according to the standard American Academy of Sleep Medicine (AASM) criteria. LM and PLM indices during sleep and wakefulness, the rate of PLMS associated with respiratory events, intermovement interval and periodicity indices were manually and automatically scored.

**Results:**

The correlation between manual and computerized scoring was high for all investigated parameters (Spearman correlation coefficients 0.751–0.996, *p* < 0.001; intraclass correlation coefficients 0.775–0.999, *p* < 0.001). Bland-Altman plots showed high agreement between manual and automatic analysis.

**Conclusions:**

This study validated an automatic LM count and PLM analysis against the gold standard manual scoring according to AASM criteria. The data demonstrate that the software used in this study has an outstanding performance for computerized LM and PLM scoring, and LM and PLM indices generated with this software can be reliably integrated in the routine PSG report. This automatic analysis is also an excellent tool for research purposes.

**Electronic supplementary material:**

The online version of this article (doi:10.1186/s12883-017-0821-6) contains supplementary material, which is available to authorized users.

## Background

Periodic leg movements (PLM) during sleep (PLMS) are present in more than 80% of patients with restless legs syndrome (RLS) [1], represent a supportive diagnostic criterium, and are associated with polymorphism in various RLS risk genes (BTBD9, TOX3/BC034767, MEIS1, MAP2K5/SKOR1, and PTPRD) [2]. They have also been observed in other sleep-related or neurological disorders, such as narcolepsy [3], sleep-related breathing disorders [4], Parkinson’s disease [5], multiple system atrophy [6] and REM sleep behaviour disorder [7], as well as in healthy subjects [8, 9].

Although several software programs for automatic detection and analysis of leg movements (LM) during sleep (LMS) have been developed and are commonly used in the clinical polysomnography (PSG) routine and research applications, only few of them have been validated and time-consuming visual detection and manual scoring of PLMS is still considered the gold standard for research purposes.

The aim of this study was to validate a LM detection and PLM analysis program integrated in a commercially available custom PSG system against manual scoring, which could be useful not only for clinical but also for research purposes.

## Methods

### Selection of participants

Routine PSG reports of the Sleep Disorders Unit, Department of Neurology, Innsbruck Medical University were screened to find 20 patients with RLS with an automatic-scored PLMS index higher than 20/h. RLS was diagnosed according to the International RLS Study Group (IRLSSG) criteria [10], based on an urge to move the legs, usually accompanied by unpleasant sensations, which begin or worsen during rest and is relieved by movement, worsen in the evening or night, and is not explained by other conditions. RLS mimics were excluded.

Control subjects were selected among patients without RLS who underwent PSG for other reasons, and had an automatic-scored PLMS index ≤ 5/h.

For both groups, exclusion criteria were an apnea-hypopnea index (AHI) higher than 5/h or the use of a continuous positive airway pressure (CPAP) therapy. RLS treatment did not represent an exclusion criterion.

This study was approved by the local ethic committee of Innsbruck Medical University. All participants granted written informed consent prior to study participation.

### Video-PSG

All subjects underwent at least one night of 8-h video-PSG according to the American Academy of Sleep Medicine (AASM) 2012 standards [11]. In case of more nights of PSG, the second night was used for this study, unless technical reasons prevented this.

Video-PSG was recorded on a OSG BrainRT PSG device (OSG 2840 Rumst, Belgium; http://www.osg.be) and consisted of electrooculography, electroencephalography (F3, F4, C3, C4, O1, O2, M1 and M2 electrodes), cardiorespiratory recording [single channel electrocardiography, recording of nasal air flow (thermocouple), nasal pressure cannula, tracheal microphone, thoracic and abdominal respiratory movements (piezo), transcutaneous oxygen saturation], electromyography (EMG) included at least the mental, submental and both anterior tibialis muscles, and time-synchronized digital videography. The video was recorded with an infrared camera (Sony IP Camera ER521P).

Leg movements were recorded using surface electrodes placed longitudinally and symmetrically around the middle of the tibialis anterior muscle, 2–3 cm apart. For scoring of EMG activity, bipolar surface EMG was recorded with the low frequency filter at 50 Hz, the high frequency filter at 300 Hz, and a sampling rate of 1000 Hz. Amplification was set at 10 μV per mm. Impedance of surface EMG electrodes had to be lower than 10 kΩ.

### Sleep and LM scoring criteria

Sleep was scored according to American Academy of Sleep Medicine (AASM) criteria [1].

According to the AASM criteria [11], the onset of a leg movement event is defined as the point at which there is an 8 μV increase in EMG voltage above resting EMG; the ending of a leg movement event is defined as the start of a period lasting at least 0.5 s during which the EMG does not exceed 2 μV above resting EMG. Bilateral leg movements separated by less than 5 s between movement onsets are counted as a single leg movement. Leg movements occurring during a period from 0.5 s preceding a respiratory event to 0.5 s following are not scored according to AASM criteria. We performed two separate analyses, one according to AASM and one including also respiratory-related leg movements.

Periodic limb movements (PLM) are defined as repetitive leg movements lasting from 0.5 to 10 s, separated by an intermovement interval (defined as the time between onsets of consecutive leg movements) ranging from 5 to 90 s, organized in series of at least 4 leg movements [11].

The periodicity index, which is an independent measure able to pick up the time structure of LM activity in patients with RLS and PLMS, was calculated as defined by Ferri et al. as the ratio of the number of periodic LM intervals (at least 3, 10–90 s) divided by the total number of intervals found [12].

### Description of manual and computerized PLM quantification and analysis

PLMS indices in NREM, REM and total sleep, PLM during wakefulness (PLMW) index, the intermovement interval in NREM, REM and total sleep, and in wakefulness, index of PLMS associated with respiratory events and periodicity index were all scored manually and automatically as explained below, and inserted in a SPSS database for the statistical analysis. The manual scoring was performed first, without running the automatic analysis, blind for the automatic scoring.

#### Manual quantification of LM and PLM

LM and PLM during sleep and during wakefulness were manually analysed by a trained scorer (AS) according to AASM criteria [1]. All identified leg movements (LM) were tabulated in an excel spread-sheet, and associated to sleep stages manually. The duration of each LM and the interval between two consecutive LM were measured. Indices were calculated for PLMS during NREM, REM and total sleep, and for PLMW. Intermovement intervals for LMS and PLMS during NREM, REM and total sleep, as well as LM during wakefulness (LMW) and PLMW, were scored. The periodicity index according to Ferri et al. was also calculated [12].

PLMS associated with respiratory events were excluded from the analysis according to the AASM standard criteria [11]. All PLMS were however recorded in the excel spread-sheet, including informations about association or not with respiratory events, so that separate analysis including or excluding PLMS associated with respiratory events were possible. Indices of PLMS associated with respiratory events per hour of total sleep time were separately calculated.

#### Computerized scoring algorithm for PLM detection and analysis

The computerized software algorithm for detection and analysis of PLM is a feature of the Brain RT PSG system by OSG (2840 Rumst, Belgium; http://www.osg.be). The algorithm was developed by OSG and adjusted in several site visits and steps upon author’s request, to automatically detect PLM according to AASM criteria.

For this study, LMS and PLMS indices in NREM, REM and total sleep, LMW and PLMW index, the intermovement interval in NREM, REM and total sleep, and in wakefulness were automatically calculated. In addition, the analysis was run again changing the settings in order to include also PLMS associated with respiratory events, and indices of PLMS associated with respiratory events per hour of total sleep time were automatically scored. No artefact correction was performed.

The analysis was then run changing in the settings the criterion for the interval between two consecutive PLMS from 5–90 s to 10–90 s, in order to be able to automatically calculate the periodicity index [12].

The settings of the software for detection and analysis of LM are shown in Additional file 1: Figure S1, and an example of the computerized detection of PLM is shown in Additional file 2: Figure S2.

### Event per event analysis

An event per event analysis was performed for each LM. All LM detected by both manual and computerized analysis were counted as matched in case of overlap between a manually detected LM and at least half of the automatically detected LM. The LMs detected only manually, as well as those detected only by computerized analysis, were also counted. The sensitivity of the computerized detection was calculated as the percentage of manually scored leg movements also detected automatically. The false positive rate was calculated as the percentage of automatically detected LM that did not match manually detected LM.

### Statistics

IBM SPSS 21 (SPSS, Inc., Chicago, IL) was used for all statistical analysis. Data were tested for normal distribution using the Shapiro-Wilk test. Descriptive statistics are given as numbers (percentages) as well as medians (range), as data were not normally distributed. Nonparametric statistics were applied. Correlations and agreement between manual and computerised quantification of LM and PLM were calculated by means of the Spearman correlation coefficients, the intraclass correlation coefficients and the Bland-Altman plots. *P*-values <0.05 were considered significant. In case of multiple comparisons, correction for Bonferroni was performed, and *p*-values were set accordingly (*p* < 0.01).

## Results

### Demographic, clinical and sleep characteristics of the RLS patients and the control group

Twenty patients with RLS (14 men, 6 women) with a median age of 51.5 (37–73) years were included in this study.

The control group included 13 men and seven women with a median age of 32 (20–60) years. The main reason for PSG examination was suspected sleep-related breathing disorder (11/20, 55%), followed by insomnia (6/20, 30%), NREM parasomnia (2/20, 10%) and suspected narcolepsy (1/20, 5%). In none of those patients sleep-related breathing disorder was confirmed by PSG, nor was narcolepsy confirmed by MSLT. The final diagnosis were primary snoring (*n* = 7), insomnia (*n* = 5), no sleep disorder (*n* = 5), NREM parasomnia (*n* = 2) and delayed sleep phase syndrome (*n* = 1). None of the patients included in the control group had relevant comorbidities, and none had central nervous system active medication.

The sleep parameters of the two groups are provided in Additional file 3: Table S1.

### Comparison of manual versus computerized detection and analysis of PLM

For this analysis, a total of 10,269 PLM (median 172.5 per subject, range 8–979) were manually scored, 6731 PLMS (median 76.5 per subject, range 1–910) and 3538 PLMW (median 44 per subject, range 0–547).

**Fig. 1:**
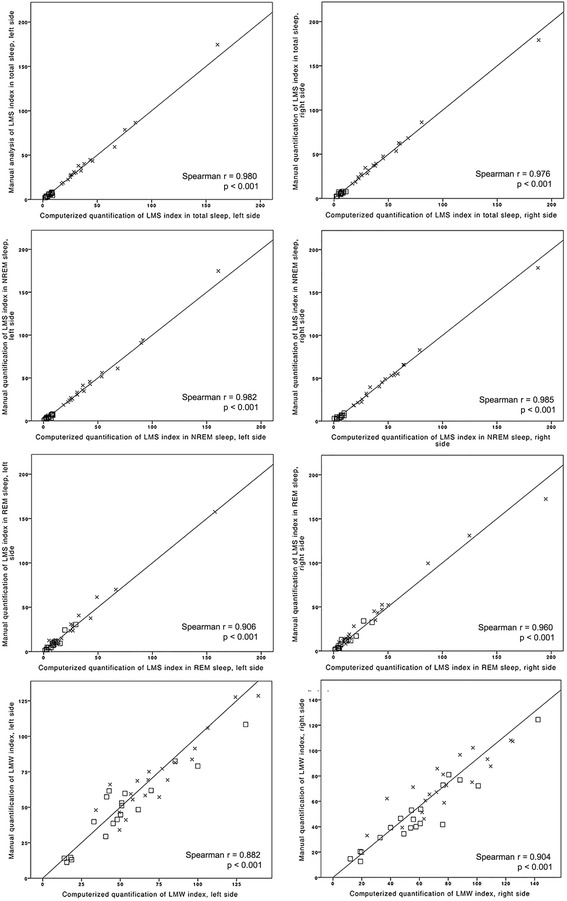
Correlations between manual and computerized scoring of LM indices. LMS, leg movements during sleep; LMW, leg movements during wakefulness. Single values for patients are represented as *crosses*, for controls as *squares*

**Fig. 2:**
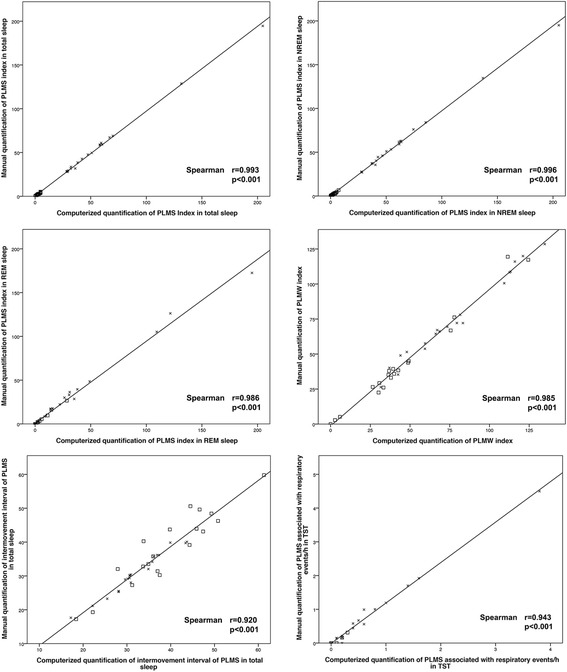
Correlations between manual and computerized scoring of PLM parameters. PLMS, periodic leg movements during sleep; PLMW, periodic leg movements during wakefulness; TST, total sleep time. Single values for patients are represented as *crosses*, for controls as *squares*

**Fig. 3:**
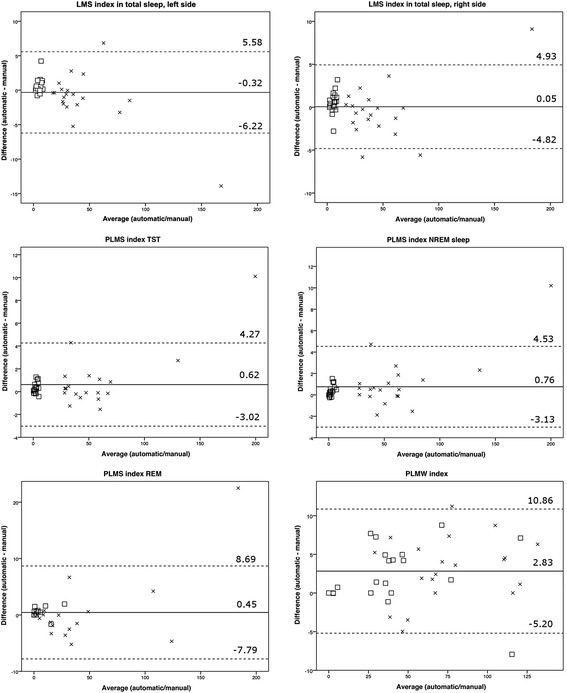
Bland-Altman plots for the different PLM indices. PLMS, periodic leg movements during sleep; TST, total sleep time; NREM, non-REM sleep; REM, rapid eye movement; PLMW, periodic leg movements during wakefulness. Single values for patients are represented as *crosses*, for controls as *squares*. The *horizontal lines* represent the mean ± 2 standard deviations (SD)

Tables 1 and 2 provide the LM and PLM measures obtained by manual and computerized detection and analysis according to AASM criteria [11], as well as the manual and computerized calculation of the periodicity index according to Ferri et al. [12] Of note, all values calculated by manual and computerized analysis were very similar. In line with this, all Spearman correlation coefficients were between 0.751 and 0.996, and all intraclass correlation coefficients between 0.775 and 0.999. Correlations between manual and computerized detection and analysis of LM and PLM are also shown in Figs. 1 and 2. The limits of agreement between manual and computerized analysis for LMS indices in total sleep (left −6.22 to 5.58, right −4.82 to 4,93), and PLMS indices in total sleep, NREM and REM sleep, and for PLMW indices, which are −3.02 to 4.27, −3.13 to 4.53, −7.79 to 8.69 and −5.20 to 10.86, respectively, are shown in Fig. 3. The mean bias was −0.32 and 0.05 for the LMS indices left and right, respectively, 0.62 for the PLMS index in TST, 0.76 for the PLMS index during NREM sleep, 0.45 for the PLMS index during REM sleep, and 2.83 for the PLMW index.

**Table 1:** Agreement between manual and computerized detection and analysis of LM, evaluated with intraclass correlation coefficients

	Manual quantification	Computerized quantification	Intraclass correlation coefficient	*P* values
LM index right				
LMS/h, TST	12.3 (6–37.4)	13.9 (6.3–37.3)	0.997 (0.995–0.999)	**<0.001** ^*^
LMS/h, NREM sleep	13.8 (4.8–44)	13.9 (5.2–43.7)	0.998 (0.996–0.999)	**<0.001** ^*^
LMS/h, REM sleep	12.8 (6.1–33.7)	11.9 (5.4–33.5)	0.989 (0.979–0.994)	**<0.001** ^*^
LMW	59.7 (39.5–80)	63.7 (48.4–86.3)	0.911 (0.839–0.952)	**<0.001** ^*^
LM index left				
LMS/h, TST	12.9 (5.4–32)	13.1 (5.4–32.1)	0.995 (0.991–0.998)	**<0.001** ^*^
LMS/h, NREM sleep	13.3 (3.8–35.7)	13.5 (4.1–36.1)	0.996 (0.992–0.998)	**<0.001** ^*^
LMS/h, REM sleep	10.5 (7–24.1)	10.3 (7–25.1)	0.991 (0.982–0.995)	**<0.001** ^*^
LMW	57.8 (42.2–76.6)	55.2 (43.9–79.6)	0.939 (0.888–0.967)	**<0.001** ^*^

*LM* leg movements, *LMS* leg movements during sleep, *LMW* leg movements during wakefulness, *TST* total sleep time

^*^Significant *p*-values after correction for multiple comparisons according to Bonferroni are given in bold letters

**Table 2:** Agreement between manual and computerized detection and analysis of PLM, evaluated with intraclass correlation coefficients

	Manual quantification	Computerized quantification	Intraclass correlation coefficient	*P* values
PLM index				
PLMS/h, TST	16.5 (0.2–194.6)	16.6 (0.2–204.7)	0.999 (0.998–0.999)	**<0.001** ^*^
PLMS/h, NREM sleep	16.9 (0–195)	17.5 (0–205.2)	0.999 (0.998–0.999)	**<0.001** ^*^
PLMS/h, REM sleep	4.4 (0–172.5)	4.4 (0–195)	0.994 (0.989–0.994)	**<0.001** ^*^
PLMW	50.2 (0–128.5)	49 (0–134.8)	0.991 (0.965–0.996)	**<0.001** ^*^
PLMS associated with respiratory events/h	0 (0–4.5)	0.1 (0–3.8)	0.977 (0.955–0.988)	**<0.001** ^*^
Intermovement interval				
TST, sec	32.8 (17.2–59.8)	34.9 (17.2–61.3)	0.945 (0.886–0.973)	**<0.001** ^*^
NREM sleep, sec	35 (17.4–59.8)	34.5 (17–61.3)	0.878 (0.778–0.875)	**<0.001** ^*^
REM sleep, sec	28.9 (15.5–62.6)	29.1 (16.2–55.2)	0.831 (0.659–0.921)	**<0.001** ^*^
Wakefulness, sec	25.4 (14.7–35.3)	22.9 (16.3–34.7)	0.779 (0.617–0.877)	**<0.001** ^*^
Periodicity index	0.8 (0–1)	0.8 (0–1)	0.958 (0.918–0.978)	**<0.001** ^*^

*PLM* periodic leg movements, *PLMS* periodic leg movements during sleep, *PLMW* periodic leg movements during wakefulness, *TST* total sleep time

^*^Significant *p*-values after correction for multiple comparisons according to Bonferroni are given in bold letters

The event per event analysis showed a high agreement between the two methods, as shown by sensitivity percentages of the computerized LM and PLM analysis ranging from 95 to 100%, and false positive percentages between 0 and 11% (Additional file 4: Table S2).

## Discussion

This study validated a software for the detection and analysis of LM integrated into a commercialy available PSG system, which quantifies different LM and PLM indices according to AASM criteria. The correlation between the computerized scoring of different LM and PLM indices and the standard visual detection and manual scoring was remarkable high. The validated integrated software allows the correlation of LM and PLM data with all other PSG data without needing to export data to another program or to run another analysis on the same platform, as the analysis can be done within the same single routine PSG report, and gives in addition visual informations. All LM and PLM parameters used not only in the routine but also for research purposes, including intermovement intervals and periodicity index, can be automatically scored with this software, and the scoring settings can be modified by the user.

Several software programs for the automatic detection and analysis of PLM are available. The first study evaluating an algorithm for automatic detection of PLM dates back to 1990 [13], and after that several algorithms for the automatic scoring of PLM were tested [14–20], but not adequately validated. To our knowledge only one [20] is included in a commercially available software for sleep analysis. However, this algorithm did not calculate intermovement intervals and periodicity index, and was not evaluated for the detection of PLMW.

Although in this study agreement between manual and automatic analysis was high for all investigated parameters, it was higher for PLM indices than for intermovement intervals. This is probably due to the fact that scoring or not scoring a few PLM makes little difference in the PLM index but can produce a sizeable difference in the intermovement interval, specifically when overall PLM indices are low. Those differences were more pronounced in REM sleep. A possible explanation is the more difficult quantification of PLM during REM sleep due to fragmentation of movements. The difference in intermovement intervals between manual and computerized scoring was more evident in the control group, which may be attributed to the reason explained above. In fact in such cases, including or not including in the analysis few PLM can produce a relevant change in intermovement intervals. Nevertheless, the software we validated showed high correlations and high agreement between both methods also in subjects with extreme PLM indices, which ranged from 0.2 to 194.6/h. The inclusion and exclusion criteria were specifically designed to validate this software in both subjects without relevant PLM and in subjects with high indices.

Of note, the periodicity indices in the current study were higher than previously reported in both RLS patients and control subjects [11]. In the original study proposing the periodicity index, Ferri and colleagues analyzed PLMS and not PLMW. Although periodicity index is based only on PLMS, including PLMW series occurring during wake after sleep onset (WASO) allows the inclusion of a higher number of PLMS that are part of mixed PLM series containing both PLMS and PLMW but constituted by <4 PLMS, that otherwise would not be counted as PLMS.

The software used for this study is fully implemented in a routine PSG system. This is on the one hand essential for the clinical routine; on the other hand, it is a time-saving system to obtain good data also for research purposes, as all the LM and PLM indices and variables can be easily calculated. No artifact correction was performed, so that the software can also be used by untrained scorers. However, the validation was done in a study with high recording quality, but manual artifact correction could be needed in recordings with worst signal quality. This software also gives the users the possibility to change several settings, with the result that it can detect and analyze LM also according different criteria.

A limitation of this study is the exclusion of patients with sleep-related breathing disorders, with or without nCPAP therapy. This should be done in further studies. Studies including different age groups, e.g. children, showing more spontaneous activity during the night, would also be of interest. Another aspect that remains to be explored is the importance of movement amplitude [21]. This was not done in this study, but amplitude measurement is possible with the software and this issue should be addressed in future studies.

As this study also validated LM count in addition to PLM analysis, these results will remain of interest also if PLM criteria will change in the future. The algorithm for LM count and PLM analysis is published, so results of the study can be replicated by other groups.

## Conclusions

The current study validated a software for the detection and analysis of LM integrated in a PSG system and commercially available against the gold standard visual detection and manual scoring according to AASM criteria, showing high agreement between both methods. The possibility to calculate several indices suggest that time-saving computerized PLM scoring is an excellent tool, useful not only in the clinical practice but also for research purposes.

## Additional files

Additional file 1: Figure S1. Settings for computerized detection and analysis of leg movements (LM) and periodic leg movements (PLM). (TIF 349 kb)
Additional file 2: Figure S2. Example of computerized detection of periodic leg movements (PLM). Legend: Leg movements are marked with green rectangles, and periodic leg movements with underlining pink bars. An overview of the PLM during the whole night is visible in the upper part of the figure, where PLM are shown as red bars. (TIF 675 kb)
Additional file 3: Table S1. Sleep parameters of the study sample. SPT, sleep period time; TST, total sleep time. SPT is defined as the elapsed time from sleep onset through the last epoch of sleep, whereas TST is the duration of time spent in NREM and REM sleep during SPT. * Significant *p*-values are given in bold letters. (DOCX 14 kb)
Additional file 4: Table S2. Sensitivity and false positive percentages of the computerized LM and PLM analysis, as derived from comparing the automated scoring algorithm to manual scoring on an event-by-event basis. Data are given as median (range) and interquartile range. *P* values are given for the comparison between RLS patients and controls. IQR, interquartile range; LM, leg movements; LMS, leg movements during sleep; LMW, leg movements during wakefulness; NREM, non-REM sleep; PLM, periodic leg movements; PLMS, periodic leg movements during sleep; PLMW, periodic leg movements during wakefulness; REM, rapid eye movement; TST, total sleep time. * Significant *p*-values after correction for multiple comparisons according to Bonferroni are given in bold letters. (DOCX 15 kb)

## Abbreviations

AASM: American Academy of Sleep Medicine
AHI: Apnoea/hypopnea index
CPAP: Continuous positive airway pressure
EMG: Electromyography
IRLSSG: International Restless Legs Syndrome Study Group
LM: Leg movements
LMS: Leg movements during sleep
LMW: Leg movements during wakefulness
PLM: Periodic leg movements
PLMS: Periodic leg movements during sleep
PLMW: Periodic leg movements during wakefulness
PSG: Polysomnography
REM: Rapid eye movement
RLS: Restless legs syndrome
WASO: Wake after sleep onset
