# Supplementary figures and images for: Validation of a leg movements count and periodic leg movements analysis in a custom polysomnography system
Source: BMC Neurol. 2017 Feb 23;17:42. doi: 10.1186/s12883-017-0821-6 (PMC5324307; doi:10.1186/s12883-017-0821-6)

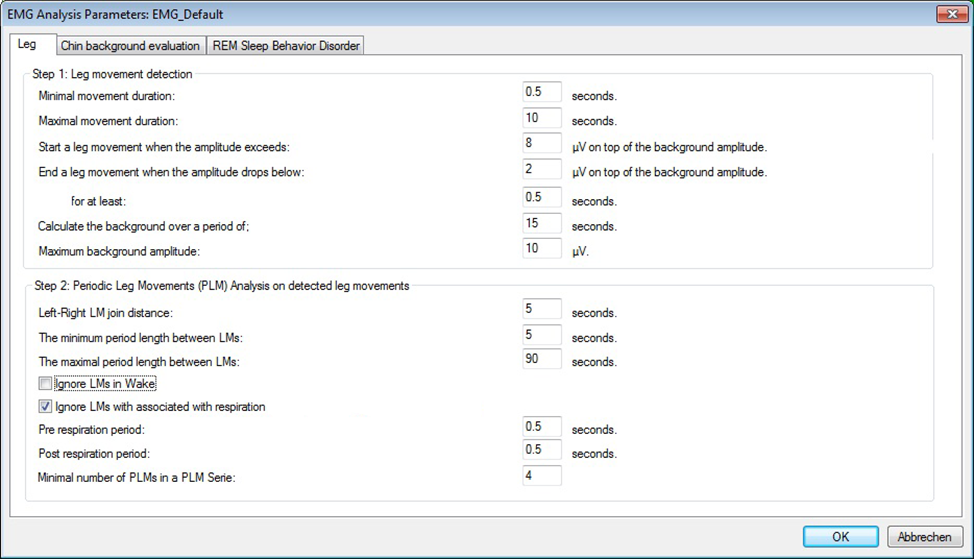

Supplement: Additional file 1: Figure S1. — Settings for computerized detection and analysis of leg movements (LM) and periodic leg movements (PLM). (TIF 349 kb) [file 12883_2017_821_MOESM1_ESM.tif]

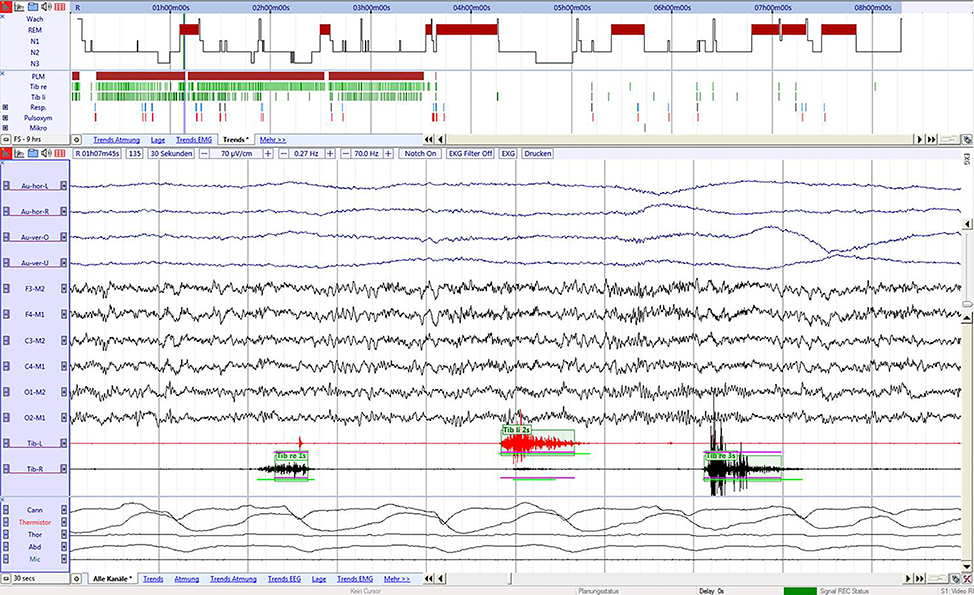

Supplement: Additional file 2: Figure S2. — Example of computerized detection of periodic leg movements (PLM). Legend: Leg movements are marked with green rectangles, and periodic leg movements with underlining pink bars. An overview of the PLM during the whole night is visible in the upper part of the figure, where PLM are shown as red bars. (TIF 675 kb) [file 12883_2017_821_MOESM2_ESM.tif]
