# Supplementary material for: Validation of a leg movements count and periodic leg movements analysis in a custom polysomnography system
Source: BMC Neurol. 2017 Feb 23;17:42. doi: 10.1186/s12883-017-0821-6 (PMC5324307; doi:10.1186/s12883-017-0821-6)
Supplement: Additional file 3: Table S1. — Sleep parameters of the study sample. SPT, sleep period time; TST, total sleep time. SPT is defined as the elapsed time from sleep onset through the last epoch of sleep, whereas TST is the duration of time spent in NREM and REM sleep during SPT. * Significant p-values are given in bold letters. (DOCX 14 kb) [file 12883_2017_821_MOESM3_ESM.docx]

**Table S1**. Sleep parameters of the study sample.

| **Sleep variables** | **RLS patients**  **N = 20** | **Control group**  **N = 20** | **P values** | |
| --- | --- | --- | --- | --- |
| **Total sleep time, min** | 408 (174 – 483) | 406.5 (345 – 458) | 0.738 | |
| **Sleep efficiency, % of SPT** | 85.6 (37.4 – 98.1) | 84.9 (70.1 – 96.6) | 0.512 | |
| **Wake after sleep onset, min** | 58 (6.5 – 259) | 41 (10 – 115) | 0.192 | |
| **Sleep stage, % of SPT**  **W**  **1**  **2**  **3**  **REM** | 12.2 (1.3 – 59.7)  11.6 (5.4 – 21.7)  51.3 (22.6 – 65.5)  4.0 (0 – 19)  19.3 (1.8 – 26.3) | 9.6 (2.2 – 25)  10.8 (6.2 – 16.1)  47.4 (31.7 – 63.1)  14.4 (0 -24.8)  18.6 (4.9 – 28.5) | | 0.183  0.289  0.758  **0.001***  0.841 |
| **Sleep onset latency, min** | 6.7 (0 – 92.9) | 9.9 (0.9 – 58.3) | 0.301 | |
| **REM sleep latency, min** | 90.3 (0.4 – 399.5) | 111.3 (49.5 – 234.5) | 0.134 | |
| **Apnea-hypopnea index, /h** | 1.5 (0 - 5) | 0.7 (0 – 4.8) | 0.023 | |

SPT, sleep period time; TST, total sleep time. SPT is defined as the elapsed time from sleep onset through the last epoch of sleep, whereas TST is the duration of time spent in NREM and REM sleep during SPT. * Significant p-values are given in bold letters.
