# Supplementary material for: Validation of a leg movements count and periodic leg movements analysis in a custom polysomnography system
Source: BMC Neurol. 2017 Feb 23;17:42. doi: 10.1186/s12883-017-0821-6 (PMC5324307; doi:10.1186/s12883-017-0821-6)
Supplement: Additional file 4: Table S2. — Sensitivity and false positive percentages of the computerized LM and PLM analysis, as derived from comparing the automated scoring algorithm to manual scoring on an event-by-event basis. Data are given as median (range) and interquartile range. P values are given for the comparison between RLS patients and controls. IQR, interquartile range; LM, leg movements; LMS, leg movements during sleep; LMW, leg movements during wakefulness; NREM, non-REM sleep; PLM, periodic leg movements; PLMS, periodic leg movements during sleep; PLMW, periodic leg movements during wakefulness; REM, rapid eye movement; TST, total sleep time. * Significant p-values after correction for multiple comparisons according to Bonferroni are given in bold letters. (DOCX 15 kb) [file 12883_2017_821_MOESM4_ESM.docx]

**Table S2.** Sensitivity and false positive percentages of the computerized LM and PLM analysis, as derived from comparing the automated scoring algorithm to manual scoring on an event-by-event basis.

|  | **Total**  **Sensitivity, %**  **False positive, %** | **RLS Patients**  **Sensitivity, %**  **False positive, %** | **Controls**  **Sensitivity, %**  **False positive, %** | **P values** |
| --- | --- | --- | --- | --- |
| **PLM** | 96 (87-100) IQR 93-97  5 (0-17) IQR 2-6 | 96 (87-100) IQR 94-97  4 (0-9) IQR 2-5 | 95 (89-1) IQR 92-98  5 (0-17) IQR 1-10 | 0.698  0.445 |
| **PLMS TST** | 97 (80-100) IQR 95-100  1 (0-24) IQR 0-3 | 97 (86-100) IQR 96-98  1 (0-8) IQR 1-4 | 100 (80-100) IQR 92-100  0 (0-24) IQR 0-2 | 0.192  0.005 |
| **PLMS NREM** | 98 (80-100) IQR 96-100  0 (0-24) IQR 0-2 | 97 (85-100) IQR 96-99  2 (0-9) IQR 1-4 | 100 (80-100) IQR 92-100  0 (0-24) IQR 0-0 | 0.041  **0.001*** |
| **PLMS REM** | 96 (70-100) IQR 89-100  0 (0-50) IQR 0-6 | 92 (83-100) IQR 89-100  4 (0-5) IQR 0-8 | 100 (70-100) IQR 86-100  0 (0-0) IQR 0-0 | 0.515  0.009 |
| **PLMW** | 94 (79-100) IQR 88-98  9 (0-20) IQR 4-13 | 93 (79-100) IQR 86-97  11 (2-20) IQR 8-14 | 97 (86-100) IQR 88-100  6 (0-15) IQR 1-13 | 0.158  0.050 |
| **LM** | 95 (73-100) IQR 93-97  6 (1-18) IQR 3-8 | 95 (88-100) IQR 93-96  4 (1-10) IQR 2-5 | 96 (73-99) IQR 93-98  8 (3-18) IQR 6-11 | 0.398  **<0.001*** |
| **LMS** | 96 (72-100) IQR 94-98  4 (0-15) IQR 2-6 | 96 (87-100) IQR 94-97  2 (0-7) IQR 1-4 | 96 (72-100) IQR 93-100  6 (0-15) IQR 4-10 | 0.461  **<0.001*** |
| **LMS NREM** | 98 (73-100) IQR 95-100  3 (0-25) IQR 1-6 | 97 (85-100) IQR 95-98  2 (0-9) IQR 1-4 | 100 (73-100) IQR 94-100  6 (0-25) IQR 2-13 | 0.221  0.021 |
| **LMS REM** | 93 (71-100) IQR 83-100  4 (0-17) IQR 0-8 | 90 (71-100) IQR 82-95  4 (0-11) IQR 0-7 | 100 (71-100) IQR 90-100  5 (0-17) IQR 0-13 | 0.035  0.513 |
| **LMW** | 92 (74-100) IQR 89-97  11 (2-25) IQR 7-14 | 92 (82-98) IQR 89-95  10 (2-19) IQR 7-13 | 96 (74-100) IQR 90-97  11 (3-25) IQR 7-14 | 0.253  0.529 |

Data are given as median (range) and interquartile range. P values are given for the comparison between RLS patients and controls. IQR, interquartile range; LM, leg movements; LMS, leg movements during sleep; LMW, leg movements during wakefulness; NREM, non-REM sleep; PLM, periodic leg movements; PLMS, periodic leg movements during sleep; PLMW, periodic leg movements during wakefulness; REM, rapid eye movement; TST, total sleep time. * Significant p-values after correction for multiple comparisons according to Bonferroni are given in bold letters.
